# Long‐term clinical outcomes of periodontal regeneration with enamel matrix derivative: A retrospective cohort study with a mean follow‐up of 10 years

**DOI:** 10.1002/JPER.21-0347

**Published:** 2021-09-08

**Authors:** Siro P. De Ry, Andrea Roccuzzo, Niklaus P. Lang, Anton Sculean, Giovanni E. Salvi

**Affiliations:** ^1^ Department of Periodontology, School of Dental Medicine University of Bern Bern Switzerland

**Keywords:** EMD, enamel matrix protein derivative, intrabony defect, long‐term results, periodontal regeneration, periodontal surgery

## Abstract

**Background:**

Despite the large body of evidence on the efficacy of enamel matrix derivative (EMD) in the treatment of periodontal intrabony defects, few studies reported long‐term data (≥10‐year).

**Methods:**

Periodontal patients treated with regenerative surgery with EMD between 1999 and 2012 were invited to participate in a clinical examination. The following clinical parameters were recorded and compared at baseline (T0), 6 months after surgery (T1) and after at least 8 years of follow‐up (T2): probing depth (PD), gingival recession (GR), clinical attachment level (CAL), plaque and bleeding scores. The primary outcome variable was CAL change.

**Results:**

Forty‐one patients with 75 treated teeth were available for analysis. Out of these, 68 (tooth survival rate: 90.7%) reached the latest follow‐up with a mean observation period of 10.3 years (range: 8.0 to 21.3). The most frequent reason for tooth loss was recurrence of periodontal disease. Tooth survival curves showed a statistically significant difference between smokers and non‐smokers (*P* = 0.028). Mean CAL changed from 8.43 ± 1.86 (T0) to 6.47 ± 1.70 (T1) (*P* < 0.001) and to 5.91 ± 1.83 (T2) (*P* < 0.001). At T1, a CAL gain of ≥3 mm was measured in 35% of the defects whereas at T2 it was detected in 51% of cases.

**Conclusions:**

Within their limitations, the present results have shown that in intrabony defects, the clinical improvements obtained following regenerative surgery with EMD can be maintained on a mean period of 10 years. Smoking status and maxillary molars were correlated with an increased risk for tooth and CAL loss, respectively.

## 1 INTRODUCTION

Periodontitis is a multifactorial inflammatory disease initiated by bacterial biofilms that may lead to progressive destruction of the tooth‐supporting apparatus and eventually to tooth loss.^1^ Even‐though in the vast majority of cases non‐surgical therapy (i.e., scaling and root planing) has been proven to be effective in terms of clinical attachment level (CAL) gain and probing depth (PD) reduction,^2^, ^3^, ^4^ some anatomical factors, such as multi‐rooted teeth with furcation involvement^5^ and the presence of deep intrabony defects,^6^ have been associated with persistent bleeding periodontal pockets and further increased risk for tooth loss.

Historically, periodontal flap surgery to gain access to the contaminated root surface has been performed to treat both supra and infrabony defects with acceptable clinical outcomes.^7^, ^8^, ^9^ Later on, with the discovery^10^ and introduction of enamel matrix derivate proteins (EMD), the paradigm for the treatment of intrabony defects has received new avenues. Indeed, EMD application was proven to result in periodontal regeneration (i.e., new periodontal ligament, new root cementum with functional periodontal ligament fibers, and new alveolar bone) in both animal^11^, ^12^ and human histological studies.^13^, ^14^, ^15^, ^16^ During the years, several clinical studies have suggested the use of EMD for the treatment of intrabony contained periodontal defects, resulting in better clinical outcomes (i.e., CAL gain and PD reduction) compared with open flap debridement procedures (OFD).^17^, ^18^, ^19^

Nevertheless, despite the large body of evidence behind the use of EMD in the short and mid‐term follow‐ups (≤5 years),^20^, ^21^, ^22^, ^23^, ^24^ only limited evidence is available on the efficacy of periodontal reconstructive procedures in association with EMD in the long‐term (i.e., ≥8 years).^25^, ^26^, ^27^, ^28^, ^29^

Therefore, the aim of the present study was to report the long‐term clinical outcomes of intrabony defects treated with EMD in patients enrolled in a supportive periodontal therapy (SPT) program up to 20‐years in the Department of Periodontology of the University of Bern, Switzerland.

## 2 MATERIALS AND METHODS

The Ethics Committee of the Canton of Bern (KEK), Switzerland, approved the study protocol (Nr. 2018‐01877). The investigation was conducted according to the revised principles of the Helsinki Declaration (2013) and a signed informed consent was obtained from each patient before beginning of the study.

### 2.1 Study sample

In this retrospective cohort study, the list of subjects diagnosed with generalized/localized chronic periodontitis according Armitage 1999^30^ who underwent periodontal surgery with EMD at the Department of Periodontology, University of Bern, Switzerland, during the period 1999 to 2012, was screened by two of the authors (S.D.R and A.R). All identified patients had been treated under senior staff supervision by graduate students as part of their educational training. The following inclusion criteria had to be met:

- Male and female patients aged ≥18 years
- Patients in systemic health or with controlled medical conditions
- Patients treated with non‐surgical periodontal therapy without systemic antibiotics completed at least 6 months prior to initiation of the reconstructive procedure
- Presence of one or more intrabony defects treated with reconstructive periodontal surgery with EMD alone
- Availability of pre‐ and postoperative patient's chart with anamnestic information, including smoking status (i.e., smoker, non‐smoker, former smoker)^31^ and complete dental treatment records
- Patients enrolled in supportive periodontal therapy (SPT) at the Department of Periodontology with full documentation of the SPT regime
- At least 8 years follow‐up after periodontal surgery

The following exclusion criteria were applied:

- Need for regenerative materials other than EMD during the surgery (i.e., bone substitutes and/or membranes)
- Intrabony defect associated with furcation involvement grade II or III
- Teeth used as abutments for fixed dental prostheses (FDPs)
- Tooth mobility degree III^32^
- Inadequate endodontic treatment and/or restoration
- Missing preoperative periodontal charts
- Patients not enrolled in a SPT program at the Department of Periodontology of the University of Bern – Switzerland
- Follow‐up period <8 years

### 2.2 Non‐surgical periodontal therapy

All subjects were treated according to a comprehensive periodontal treatment plan.^33^ Following case presentation and oral hygiene instruction, non‐surgical periodontal therapy was carried out under local anesthesia without the prescription of systemic antibiotics. Approximately 3 months after the completion of this phase, all patients underwent a periodontal re‐examination with full periodontal charts and whenever needed subgingival re‐instrumentation was performed. After a healing phase of 3 additional months following periodontal re‐evaluation and re‐instrumentation, patients displaying teeth with persisting PD ≥6 mm were scheduled for periodontal surgery. All teeth exhibiting increased mobility (i.e., grade 2) were splinted prior to surgery.^34^

### 2.3 Surgical regenerative procedure and postoperative care

All patients underwent the same surgical and post‐surgical protocol. Under local anesthesia full thickness, mucoperiosteal flaps were raised by means of modified or simplified papilla preservation techniques (MPPT and SPPT)^35^, ^36^ according to the mesio‐distal width of the interproximal space to access the intrabony defect area. Following removal of granulation tissue, roots were scaled and planed using Gracey curettes and an ultrasonic device. Additionally, the debrided root surfaces were conditioned with 24% EDTA‐Gel* for 2 minutes followed by a 1 minute rinse with sterile saline solution. Then, after careful root surface drying with sterile gauzes, EMD† was applied on the root surface. Finally, the mucoperiosteal flaps were adapted to allow for primary and tension‐free closure with vertical or horizontal mattress sutures.

Postoperatively, patients were instructed to rinse twice daily with a 0.2% chlorhexidine digluconate rinsing solution for 60 seconds for 2 weeks. Patients were instructed to take non‐steroidal analgesics (NSAID), as needed, and were advised to discontinue tooth and interproximal brushing in the areas of surgery and to avoid chewing trauma for 3 weeks. Sutures were removed no earlier than 14 days after surgery. Supragingival prophylactic procedures without subgingival probing and instrumentation were scheduled every second week during the first 2 months postoperatively.

After at least 6 months of healing phase, patients were enrolled in a SPT program with a recall frequency of 3 months in the first year.

### 2.4 Supportive periodontal therapy (SPT)

After the first year, all patients were enrolled in an individualized SPT program. More specifically, all patients were seen 3 to 4 times per year according to the individual needs. During the entire observation period, recall appointments, consisting of oral hygiene reinforcement and supra‐ and subgingival tooth cleaning were performed by experienced dental hygienists.

Compliance with SPT was calculated by dividing the number of recall visits attended by the patient with the number of planned appointments and expressed in percentage.

### 2.5 Clinical assessment

Approximately 6 months following non‐surgical periodontal therapy (T0), 6 months after periodontal regenerative surgery (T1) and at the latest follow‐up examination, performed between January 2019 and December 2020, (T2) the clinical parameters were recorded around each treated tooth by means of a XP23/UNC 15 probe.‡ At T0 and T1 all the measurements were recorded by the same dentist who had performed the periodontal treatment, whereas at the latest follow‐up (T2), the clinical assessment was performed by one of the authors (S.D.R) not involved in any part of the treatment. The following clinical variables were recorded at six sites per tooth (i.e., mesio‐buccally (mb), mid‐buccally (b), disto‐buccally (db), mesio‐lingually (ml), mid‐lingually (l) and disto‐lingually (dl)):

- Clinical attachment level (CAL): distance in millimeters from the cemento‐enamel junction (CEJ) to the bottom of the pocket.
- Probing depth (PD): distance in millimeters from the gingival margin to the bottom of the pocket.
- Gingival recession (GR): distance in millimeters from the gingival margin to the cemento‐enamel junction (CEJ).
- Presence or absence of dental plaque (PI).^37^
- Presence or absence of bleeding on probing (BoP).^38^

In addition, for each patient the following full‐mouth periodontal variables were recorded:

- Full‐mouth plaque score (FMPS)^37^: percentage of tooth sites revealing the presence of dental biofilms;
- Full‐mouth bleeding score (FMBS)^38^: percentage of tooth sites revealing the presence of bleeding on probing

Data were reported in accordance with the STROBE checklist.^39^

### 2.6 Statistical analysis

All statistical analyses were performed using IBM SPSS Statistics for Windows, version 26.0.0.0.§

The deepest PD measure at T0 (i.e., baseline) of each treated tooth was considered. The same site was measured at the follow‐up time points (i.e., T1 and T2). Descriptive statistics were expressed using means with standard deviation (SD) and ranges for continuous variables and relative frequencies (%) for categorical variables. Additionally, 95% confidence intervals (CI) were calculated for mean differences of parameters between time‐points.

ANOVA of repeated measurements was conducted to analyze changes with respect to FMPS and FMBS over time, attending to Bonferroni's correction in multiple pairwise comparisons. Linear models of repeated measurements using generalized estimation equations (GEE) were performed to analyze changes over time of parameters measured at tooth‐level, (i.e., PD, GR, CAL, BoP, and PI) because of the within‐subjects dependence of observations. The effect of smoking on tooth loss was analyzed using binary logistic regression from GEE models. However, non‐parametric tests (i.e., Kruskal‐Wallis) were used to analyze differences in distribution of tooth loss through different tooth types instead of logit approach because the lack of convergence. The effect of smoking and tooth type on changes of CAL and PD over time was investigated by GEE models including interaction terms.

Survival Kaplan‐Meier analysis was conducted to analyze time to events for tooth and CAL loss >2 mm, respectively and log‐rank test was used to compare survival curves between levels of factors. All tests were two tailed and *P* values <0.05 were defined as statistically significant.

## 3 RESULTS

### 3.1 Patient and defect characteristics

Five‐hundred and forty‐eight patients underwent periodontal regenerative therapy between January 1999 and December 2012 at the Department of Periodontology, University of Bern, Switzerland. After identification and screening, 57 patients fulfilling the inclusion criteria were invited to take part in the study. Finally, data from 41 patients were collected and used for analysis (see Supplementary Figure S1 in online *Journal of Periodontology*).

The present cohort included forty‐one patients (20 women and 21 men), with a mean age of 49.0 ± 11.4 years (range: 23 to 77) at baseline (i.e., re‐evaluation 6 months after non‐surgical periodontal therapy) (T0), who underwent periodontal regenerative surgery with EMD alone. Each patient contributed with at least one test tooth for a total of 75 intrabony defects.

Twelve patients (29.2%) were smokers of <20 cigarettes per day, nine subjects (22.0%) former‐smokers whereas 20 patients (48.8%) were non‐smokers.

At T0, the mean number of teeth per patient was 24.2 ± 3.7 (range: 16 to 32), whereas the mean number of teeth with PD >5 mm was 5.7 ± 3.2 (range: 1 to 13). Mean FMPS and FMBS were 17.6% ± 9.8 (range: 1 to 48%) and 19.5% ± 10.1 (range: 5 to 44%), respectively.

The 75 intrabony defects were in 57.3% (n = 43) in the maxilla and 42.7% (*n *= 32) in the mandible. More specifically, the most frequently treated tooth type was the premolar (*n* = 28; 37.3%). Details of patients’ and defects characteristics are summarized in Table 1 and Supplementary Table S1 in the online *Journal of Periodontology*.

**TABLE 1:** Pre‐treatment patient, teeth, and defect characteristics

	*n* (%)	Mean ± SD	Range
N patients	41		
N teeth	75		
Age		49.0 ± 11.4	23‐77
Smoking status			
Non‐smoker	20 (48.8)		
Smoker	12 (29.2)		
Former smoker	9 (22.0)		
FMPS (%)		17.6 ± 9.8	1‐48
FMBS (%)		19.5 ± 10.1	5‐44
Full‐mouth N of teeth		24.2 ± 3.7	16‐32
N of teeth with PD >5 mm		5.7 ± 3.2	1‐13

### 3.2 Tooth survival analysis

**FIGURE 1:**
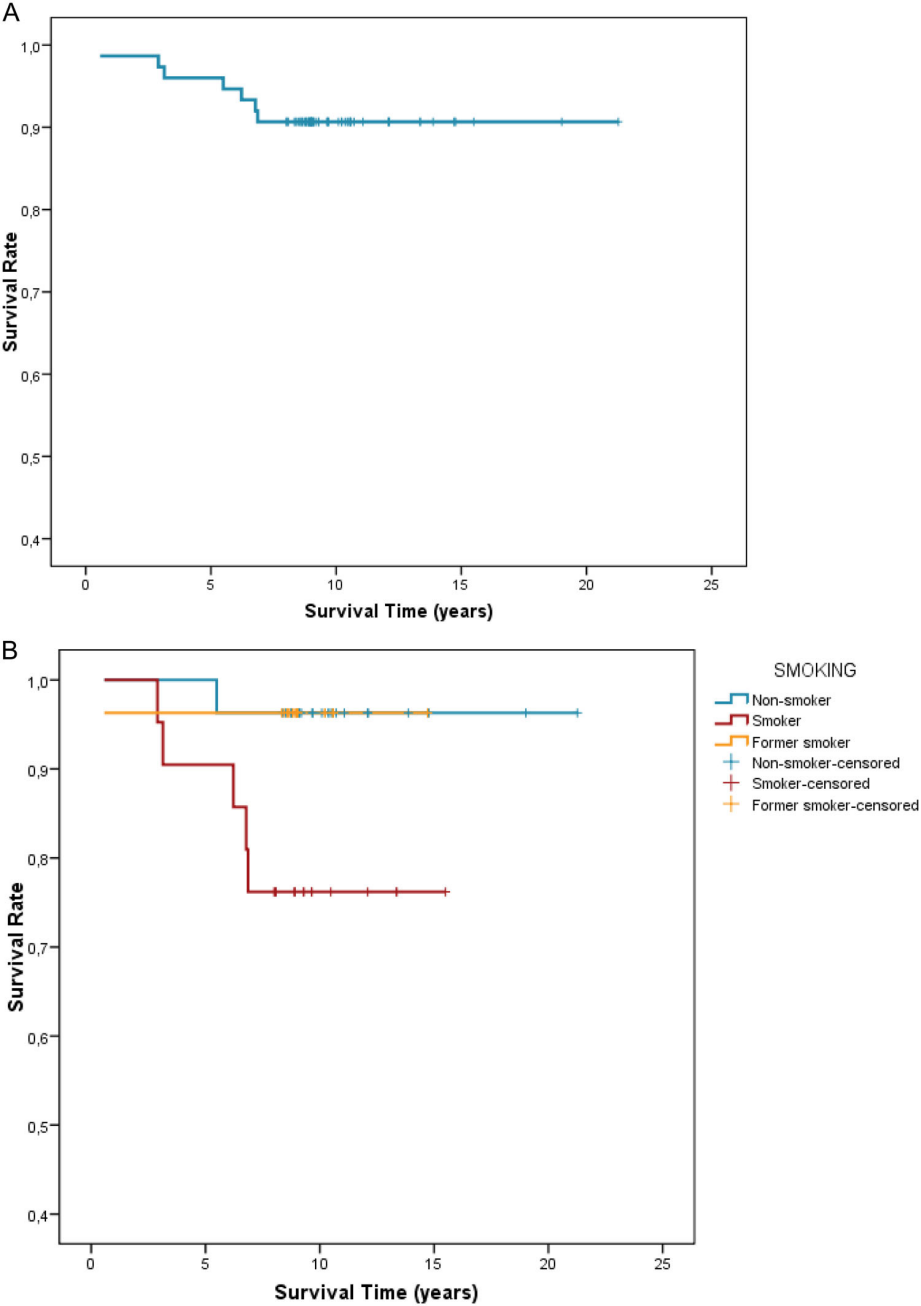
(**A**) Kaplan‐Meier for survival in terms of non‐avulsion. (**B**) Kaplan‐Meier for survival in terms of non‐avulsion in smokers, non‐smokers, and former‐smokers

Of the 75 teeth surgically treated, seven teeth were extracted yielding an overall survival rate of 90.7%. With respect to the remaining 68 teeth, the latest follow‐up was performed after a mean period of 10.3 years (range: 8.0 to 21.3). Five out of the seven extracted teeth were in smokers (23.8%), whereas in non‐smokers and former smokers only one tooth per group was extracted (3.7%). This difference did not reach statistical significance (*P* = 0.056) as well as the calculated OR (8.13) for tooth loss in smokers (*P* = 0. 062) (CI 95%: 0.9 to 73.4). The survival curves for the entire cohort and for smokers, non‐smokers and former‐smokers are illustrated in Figure 1A‐B. More specifically, survival curves showed a statistically significant difference (*P* = 0.028) according to smoking status.

The most prevalent cause of tooth extraction was periodontal disease progression (n = 3). Details of the teeth requiring extraction are reported in Table 2.

**TABLE 2:** List of reasons for tooth loss

				Values in the deepest site							
Patient #	Tooth #	Smoking	Compliance (%)	PD T0 (mm)	GR T0 (mm)	CAL T0 (mm)	PD T1 (mm)	GR T1 (mm)	CAL T1 (mm)	Years of Extraction	Reason
3	24	Yes	78	8	0	8	7	2	9	2.9	Perio
4	14	Yes	95	7	3	10	5	4	9	6.2	Perio
8	24	No	100	9	0	9	5	1	6	5.5	Perio‐endo lesion
14	35	Yes	92	6	2	8	6	2	8	3.1	Root fracture
14	45	Yes	92	6	1	7	4	2	6	6.8	Perio
27	26	Yes	78	6	3	9	5	3	8	6.9	Perio‐endo lesion
37	26	Former	82	6	2	8	3	4	7	0.6	Root fracture

### 3.3 Clinical parameters: patient‐based analysis

The means for FMBS and FMPS at the three time points are summarized in Table 3A. Low mean FMPS were recorded at both T0 (17.7% ± 10.2) and T1 (13.4% ± 7.0), whereas a statistically significant increase of full‐mouth plaque accumulation was detected at T2 (24.9% ± 12.3) (*P* < 0.001). When focusing on FMBS, a statistically significant difference between T0 (20.3% ± 10.0) and T1 (9.2% ± 5.7) was detected (*P* < 0.001) whereas a comparable percentage (9.2% ± 7.4) was recorded at the latest follow‐up (T2) (*P* = 1.000).

**TABLE 3:** (**A**) Full‐mouth plaque scores (FMPS) and full‐mouth bleeding scores (FMBS) at baseline, at re‐evaluation after periodontal regeneration and latest follow‐up (means or percentages ± SD) (*n* = 38). (**B**) Mean clinical parameters and frequency distribution (%) of BoP and plaque at the deepest site of the treated teeth which reached the latest follow‐up examination (means ± SD) (*n* = 68)

				Mean difference (95% CI) and *P*‐value		
	T0	T1	T2	T0 versus T1	T1 versus T2	T0 versus T2
**(A)**						
FMPS (%)	17.7 ± 10.2	13.4 ± 7.0	24.9 ± 12.3	−4.3 (−7.8 −0.8) *P* = 0.012	11.5 (6.7 16.2) *P* < 0.001	7.2 (2.7 11.7) *P* = 0.001
FMBS (%)	20.3 ± 10.0	9.2 ± 5.7	9.2 ± 7.4	−11.1 (−15.5 −6.8) *P* < 0.001	0.0 (−3.5 3.5) *P* = 1.000	−11.1 (−15.7 −6.6) *P* < 0.001
**(B)**						
PPD (mm)	6.71 ± 1.22	3.78 ± 1.24	3.75 ± 1.41	−2.93 (−3.25 −2.60) *P* < 0.001	−0.03 (−0.47 0.41) *P* = 0.896	−2.96 (−3.46 −2.45) *P* < 0.001
GR (mm)	1.72 ± 1.18	2.69 ± 1.21	2.16 ± 1.62	0.97 (0.68 −1.26) *P* < 0.001	−0.53 (−0.83 −0.23) *P* < 0.001	0.44 (0.10 −0.78) *P* = 0.011
CAL (mm)	8.43 ± 1.86	6.47 ± 1.70	5.91 ± 1.83	−1.96 (−2.32 −1.59) *P* < 0.001	−0.56 (−1.02 −0.09) *P* = 0.018	−2.52 (−3.06 −1.97) *P* < 0.001
BoP + (%)	73.5	30.9	38.2	−43.6 (−57.2 28.1) *P* < 0.001	7.4 (−9.0 −23.7) *P* = 0.379	−35.3 (−51.2 −19.3) *P* < 0.001
BoP – (%)	26.5	69.1	61.8	–	–	–
PI + (%)	36.8	19.1	45.6	−17.6 (−29.6 −0.06) *P* = 0.004	26.5 (14.3 38.6) *P* < 0.001	8.8 (−7.6 25.2) *P* = 0.291
PI – (%)	63.2	80.9	54.4	–	–	–

### 3.4 Clinical parameters: site‐based analysis

The site based (*n* = 68) PD, GR, and CAL values at T0, T1 and T2 are shown in Table 3B.

At T1 (i.e., 6‐months after surgery), a statistically significant mean decrease in PD of 2.93 mm was detected compared to T0 (i.e., from 6.71 mm ± 1.22 to 3.78 mm ± 1.24) (*P* < 0.001). At the latest follow‐up (T2), mean PD was still statistically significantly reduced compared to baseline (*P* < 0.001), and did not differ between T1 and T2 (PD change 0.03 mm; *P* = 0.896).

Both at T1 and T2, the mean GR was statistically significantly increased compared to baseline (i.e., 0.97 mm at T1; *P* < 0.001 and 0.44 mm at T2; *P* = 0.01). Between T1 and T2, a statistically significant reduction in the mean GR depth was detected (i.e., ‐0.53 mm; *P* < 0.001).

Mean CAL change demonstrated statistically significant improvements at T1 (‐1.96 mm) as well as at T2 (‐2.52 mm) compared with T0 (*P* < 0.001). Finally, a statistically significant difference in mean CAL change between T1 and T2 was also detected (‐0.56 mm; *P* = 0.018).

When analyzing the mean CAL and PD change through time with respect to tooth type (i.e., incisor/canine; premolar, molar) and location (i.e., maxillary or mandibular), a statistically significant difference was detected (*P* < 0.001).

**FIGURE 2:**
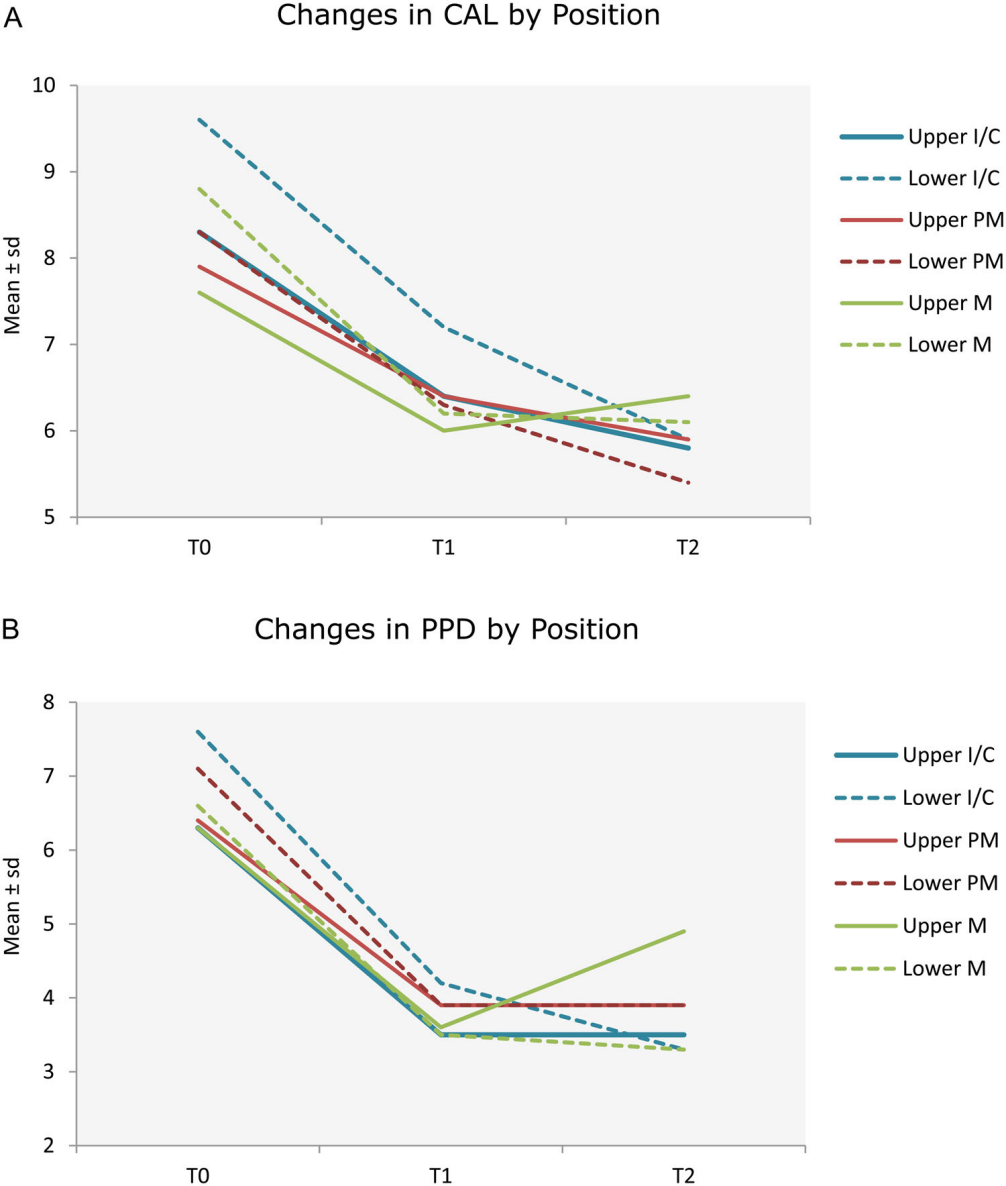
(**A**) Changes in CAL in the deepest site by tooth type. C, canines; I, incisors; M, molar; PM, premolar. (**B**) Changes in PD in the deepest site by tooth type

In particular, between T1 and T2, all teeth with the exception of upper molars (i.e., green continuous line) displayed a slight mean CAL gain. A similar negative trend was detected in upper molars with an increase in PD of 1.33 mm through time when compared to all the other tooth‐types. Details of the CAL and PD changes through time are reported in Figure 2A‐B.

The mean BoP and PI values at the treated sites recorded at the three time points are summarized in Table 3B. A great improvement in BoP values was detected after surgery (i.e., from 73.5% to 30.9%; *P* < 0.001) whereas no statistically significant difference was detected between T1 and T2 (*P* = 0.379). With respect to PI scores, statistically significantly higher scores were detected at T2 compared to T1 (i.e., from 19.1% to 45.6%; *P* < 0.001).

The frequency distributions of CAL gain at T1 and T2 are depicted in Table 4.

**TABLE 4:** (**A**) Frequency distribution (n; %) of CAL gain at re‐evaluation after periodontal surgery and at the latest follow‐up evaluation of treatment outcomes according to either “conventional” probing measurements or COM outcome. (**B**) Evaluation of treatment outcomes according to either “conventional” clinical measurements (i.e., CAL, PD) or COM outcome

**(A)**	T1	T2
CAL gain (mm)		
<0 (loss)	3 (4.4)	5 (7.4)
0‐2	41 (60.3)	28 (41.2)
3	15 (22.1)	15 (22.1)
4	6 (8.8)	11 (16.2)
5	2 (2.9)	3 (4.4)
>5	1 (1.5)	6 (8.8)
Total	68 (100)	68 (100)
**(B)**		
CAL change (mm)	mean ± SD: 1.96 ± 1.49 median (IR): 2.00 (1‐3) min‐max range: ‐1 – 6	mean ± SD: 2.51 ± 2.10 median (IR): 3.00 (1‐4) min‐max range: ‐2 – 8
PD (mm)	mean ± SD: 3.78 ± 1.24 median (IR): 4.00 (3‐4) min‐max range: 2 – 7	mean ± SD: 3.75 ± 1.41 median (IR): 4.00 (3‐5) min‐max range: 1 – 8
COM		
CAL gain ≥ 3 mm & PD ≤ 4 mm (success) CAL gain ≥ 3 mm & PD > 4 mm CAL gain < 3 mm & PD ≤ 4 mm CAL gain < 3 mm & PD > 4 mm (failure)	30.9% 4.4% 45.6% 19.1%	42.6% 8.8% 30.9% 17.6%

At T1, a CAL gain of ≥3 mm was measured in 35% of the defects (i.e., 24 of 68), whereas at the latest follow‐up examination, it was detected in 51% of cases (i.e., 35 of 68). Finally, applying the Composite Outcome Measure (COM) score^40^ to evaluate the efficacy of periodontal regenerative procedures, success was detected in 30.9% and 42.6% of cases at T1 and T2, respectively.

## 4 DISCUSSION

The present study indicated that regenerative periodontal therapy of intrabony defects using EMD alone yielded positive clinical outcomes that could be successfully maintained both in the short (T1) as well as in the long‐term (T2) up to 21.3 years.

The additional benefit of regenerative surgical procedures to treat intrabony defects compared to open flap debridement (OFD) procedures has been mainly investigated in the short term.^17^, ^18^, ^19^ Nevertheless, a study by Cortellini,^41^ reported the favorable long‐term outcomes of periodontal regeneration with GTR in intrabony defects followed by 20 years of SPT highlighting the long‐term benefits of such treatment approach.

When focusing on the 1‐year clinical outcomes of this study, it has to be noted that the use of EMD resulted in statistically significant improvements in PD and CAL compared to baseline. This finding is in accordance with available evidence reported in two systematic reviews.^42^, ^43^ Nevertheless, it has to be underlined that the magnitude of the short‐term CAL gain in the present study (i.e., 1.96 mm) is slightly lower than that reported in the literature (range: 2.0 to 4.7 mm).^17^, ^19^, ^44^, ^45^, ^46^, ^47^, ^48^, ^49^, ^50^, ^51^ One possible explanation might be the lower baseline PD (i.e., 6.71 mm) in the present study compared to the ones previously reported ranging from 7.5 to 9.6 mm.^47^, ^52^ Indeed, it is known that the magnitude of the CAL gain after periodontal treatment is directly correlated to the initial PD value.^7^

On the other hand, the long‐term mean CAL gain of 2.52 mm in the present study is comparable with previous publications reporting a mean CAL gain ranging from 2.6 to 3.0 mm.^21^, ^26^

When evaluating the overall treatment success applying the composite outcome (i.e., CAL + PD) proposed by Trombelli et al. 2020, a success rate of 30.9% at 6 months (T1) and of 42.6% after a mean of 10.3 years (T2) was detected. Our results are similar to those reported by Trombelli et al. 2020 who reported an overall of treatment success of 41.5%.

When stratifying per tooth type, it is interesting to note, that all tooth types displayed a stability or an additional improvement with respect to mean CAL values during follow‐up except for upper molars experiencing a deterioration (i.e., green line on Figure 2A). This deterioration was also detected when analyzing PD values, with a mean increase between T1 and T2 in upper molars from 3.56 to 4.89 mm (*P* < 0.001).^53^

Out of the 75 teeth available for analysis, 7 were extracted. Periodontal disease progression was the most prevalent cause of tooth extraction (n = 3; 43%). This finding indicates a tooth survival rate of 90.7% after 10 years of SPT. It is interesting to note that the majority of tooth loss (71%) and all teeth lost because of periodontal disease recurrence (100%) was observed in smokers.

Smoking has been demonstrated to influence the outcome of regenerative periodontal treatment.^54^, ^55^, ^56^ However, all the above listed studies used classic GTR procedures by means of non‐resorbable (e‐PTFE) or resorbable (polylactic acid) membranes. Only few clinical studies^57^, ^58^ investigated the reliability of EMD alone in patients with different smoking status. The present findings corroborate the findings of Heijl et al. (1997)^57^ indicating that smokers experienced more tooth loss than non‐smokers and previous smokers. Although the difference with respect to tooth loss between smokers and non‐smokers failed to reach statistical significance in the present study, a tendency is indisputable owing to the underpowered material.

Despite the provided non‐surgical and/or surgical periodontal treatment, patients’ adhesion to a regular SPT program has been demonstrated to be of paramount importance to maintain the obtained long‐term results.^3^, ^31^, ^59^ In the present study, all patients were seen 3 to 4 times per year, based on their risk profile calculated at the end of active periodontal treatment,^60^ resulting in an overall mean percentage of patients’ adhesion to the SPT program of 86%. This finding is corroborated by the overall low FMPS and FMBS recorded at the three follow‐up examinations. Our findings are consistent with previous results indicating that the positive clinical outcomes after periodontal regenerative procedures could only be maintained in patients enrolled in a strict SPT regime.^61^, ^62^

Despite several data have pointed out that clinical outcomes after periodontal regenerative procedures might be influenced by the clinical experience of the surgeon,^18^, ^46^, ^63^ the present results indicated that the surgical application of EMD might also be successfully used by postgraduate students, providing external validity of the obtained data.

The present study has several limitations. Firstly, the retrospective study design and the limited sample size affected the obtained outcomes. Second, the lack of a control group with intrabony defects treated with OFD alone does not allow any definitive conclusion. This means that the outcomes of the present study cannot be compared with outcomes of OFD alone or other regenerative procedures such as GTR. In addition, the lack of precise intra‐operative assessment of the intrabony defect configuration as well as the assessment of the clinical parameters by different operators over a long period of time ranging from 8 to 21 years and the lack of radiographic evaluation represent limitations in clinical reproducibility. Nevertheless, it must be mentioned that the majority of the defects were contained‐type defects with a 2 to 3 wall intraosseous component.

With respect to smoking status, it must be pointed out that patients’ self‐reported data on their habit remains still questionable.^64^ In addition, smoking status was assessed only once before periodontal surgery and, hence, it cannot be excluded that during the observation period patients’ habits might have changed, affecting periodontal conditions. Based on all the reasons listed above the present data should be interpreted with caution.

## 5 CONCLUSION

In conclusion, within their limitations, the present results indicated that in intrabony defects, the clinical improvements obtained following regenerative therapy with EMD alone can be successfully maintained over a mean period of 10 years. Smoking status and maxillary molars were correlated with an increased risk for tooth and CAL loss, respectively.

## CONFLICTS OF INTEREST

The authors declare no conflicts of interest.

## AUTHOR CONTRIBUTIONS

S.D.R., A.R. conceived the idea; S.D.R, A.R. collected, analyzed the data, contributed to the writing; N.P.L., G.E.S., A.S. contributed to the writing and critically revised the manuscript. All authors agreed on the final manuscript draft.

## Supporting information


**SUPPLEMENTARY FIGURE 1** Flow‐chart of the identified, re‐evaluated and analyzed patients

**SUPPLEMENTARY TABLE 1**. Frequency distribution of the defects (n; %) according to jaw and tooth type

## Footnotes

* PrefGel, Institut Straumann AG, Basel, Switzerland.

† Emdogain, Institut Straumann AG, Basel, Switzerland.

‡ Hu‐Friedy, Chicago, IL.

§ IBM Corp., Armonk, NY.
