# Supplementary material for: Long‐term clinical outcomes of periodontal regeneration with enamel matrix derivative: A retrospective cohort study with a mean follow‐up of 10 years
Source: J Periodontol. 2021 Sep 8;93(4):548–59. doi: 10.1002/JPER.21-0347 (PMC9373923; doi:10.1002/JPER.21-0347)
Supplement: Supplementary file 1 — SUPPLEMENTARY FIGURE 1 Flow‐chart of the identified, re‐evaluated and analyzed patients [file JPER-93-548-s002.docx]

**SUPPLEMENTARY FIGURE 1** Flow-chart of the identified, re-evaluated and analyzed patients

**Identification and screening**

Patients excluded (n=491)

Reason for exclusion:

- use of biomaterials other than EMD (n=186)
- furcation involvement grade II or III (n=109)
- SPT in private practice (n=173)
- patients with missing documentation (n=6)
- deceased (n = 17)

Patients underwent regenerative periodontal therapy between 1999 and 2012

(n = 548)

Patients assessed for eligibility
(n = 57)

**Re-evaluation**

Patients excluded (n=16)

Reason for exclusion:

- unwilling to attend re-evaluation (n=16)

**Inclusion**

Patients included in the analysis
(n = 41)
