# Supplementary material for: Long‐term clinical outcomes of periodontal regeneration with enamel matrix derivative: A retrospective cohort study with a mean follow‐up of 10 years
Source: J Periodontol. 2021 Sep 8;93(4):548–59. doi: 10.1002/JPER.21-0347 (PMC9373923; doi:10.1002/JPER.21-0347)
Supplement: Supplementary file 2 — SUPPLEMENTARY TABLE 1. Frequency distribution of the defects (n; %) according to jaw and tooth type [file JPER-93-548-s001.docx]

**SUPPLEMENTARY TABLE 1.** Frequency distribution of the defects (n; %) according to jaw and tooth type

|  | **Maxilla (%)** | **Mandible (%)** | **Total (%)** |
| --- | --- | --- | --- |
| Incisor/canine | 13 (17.3) | 13 (17.3) | 26 (34.7) |
| Premolar | 19 (25.3) | 9 (12.0) | 28 (37.3) |
| Molar | 11 (14.7) | 10 (13.3) | 21 (28.0) |
| *Total* | 43 (57.3) | 32 (42.7) | 75 (100) |
